# Lifting the Mouth Corner: A Systematic Review of Techniques, Clinical Outcomes, and Patient Satisfaction

**DOI:** 10.1093/asj/sjac077

**Published:** 2022-04-04

**Authors:** Nanouk van der Sluis, Haydar A Gülbitti, Joris A van Dongen, Berend van der Lei

**Affiliations:** Department of Plastic Surgery, University of Groningen, University Medical Center Groningen, Groningen, the Netherlands; Department of Dentistry, University of Groningen, University Medical Center Groningen, Groningen, the Netherlands; Department of Plastic, Reconstructive, and Hand Surgery, Utrecht University Medical Center, Utrecht University, Utrecht, the Netherlands; Department of Plastic Surgery, University of Groningen, University Medical Center Groningen, Groningen, the Netherlands

## Abstract

**Background:**

Mouth corners are an essential part of the centrofacial area for perception of attractiveness and emotions. Downturned mouth corners are a result of aging or have a congenital origin. Different mouth corner lifting techniques are described in the literature.

**Objectives:**

This review was performed to systematically assess and compare invasive and noninvasive mouth corner lifting techniques and their effectiveness, patient satisfaction, and adverse effects.

**Methods:**

MEDLINE (via PubMed), EMBASE (OvidSP), and the Cochrane Central Register of controlled trials databases were searched for clinical and observational studies published in peer-reviewed academic journals with abstracts available (searched from May 18, 2019, to December 18, 2021). Outcomes of interest were aesthetic mouth corner lifting techniques, the degree of lift as well as the longevity of the lifting effect, patient satisfaction, and adverse effects. Techniques were subdivided in invasive techniques and noninvasive techniques.

**Results:**

Out of 968 studies found from the search, 11 were included in the qualitative analysis. In general, surgical techniques seem to have a better mouth corner lifting effect than nonsurgical techniques; however, objective evidence is weak, and surgery inevitably results in a scar. Reported patient satisfaction was good for both surgical and nonsurgical techniques and no severe complications have been described.

**Conclusions:**

Surgical techniques seem to have a better lifting effect on mouth corners than nonsurgical techniques. Nevertheless, objective evidence is weak, and a scar is inevitable.

**Level of Evidence: 4:**

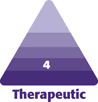

See the Commentary on this article here.

“Beauty is around the eyes and lips” was demonstrated by anthropologists in the 1990s^1^ and by psychologists by means of eye-tracking studies.^2^ People mainly look to the centrofacial area (the area around the eyes, nose, and lips), which determines facial attractiveness and beauty.^1-3^ An attractive mouth is characterized by full lips, smooth skin without folds, and an upward position of the oral commissures (mouth corners). An upward position of the oral commissures is associated with an impression of pleasure and amiability, whereas a depressed position of the oral commissures is associated with sadness, anger, and seriousness.^4,5^ Furthermore, drooping mouth corners create a natural pathway for saliva to escape down the marionette lines.^6^

The interaction of the perioral muscles affects the position of the oral commissures. During aging, the oral commissures depress over time partly due to increased contractility of the depressor anguli oris (DAO) muscles, sagging of facial fat pads (jowling), and deterioration of skin smoothness.^7^ Downturned oral commissures could also have a congenital origin, which seems to result from hyperactive DAO and mentalis muscles or an imbalance of mouth corner elevators and depressors^6,8^

Various techniques to lift the oral commissure have been described in the literature.^9^ Initially, in the early 1960s, surgical interventions began to be performed to lift mouth corners.^10-14^ At that time, indications to lift oral commissures were mainly posttrauma surgery or after tumor excision. Lifting the oral commissures for aesthetic purposes gained more interest from the end of the 20th century.^15-18^ At the beginning of the 21st century, noninvasive techniques, eg, injection with fillers and neurotoxins, also started to be developed.

Nowadays, to lift or rejuvenate oral commissures both surgical techniques (eg, Z-plasty,^19^ lentiform excision,^20^ triangular excision,^21^ a triangular excision combined with a subnasal lift; ^22^ or more extended methods, eg, a combination of an incision with DAO transection^6,22,23^) and nonsurgical techniques (eg, injecting botulinum toxin [Botox, Allergan, Irvine, CA], fillers or a combination of these^24^) have been described in the literature. Perkins et al provided an overview of the indications and (dis)advantages of surgical mouth corner lifting procedures.^9^ Nonetheless, it remains unclear which surgical technique results in the optimum mouth corner lift and whether surgical techniques lead to a more evident mouth corner lift than nonsurgical techniques. Therefore, the aim of this study was to systematically assess both invasive and noninvasive mouth corner lifting techniques described in the literature, while including efficacy, patient satisfaction, and adverse effects.

## METHODS

### Protocol and Registration

This systematic review was performed following the PRISMA guidelines.^25^ The search strategy was based on a population, intervention, comparison, outcome (PICO) framework.^26^ This study was not registered.

### Eligibility Criteria

Studies were included when at least 1 invasive or noninvasive surgical technique was used to lift the mouth corners. Studies were excluded when the effect on the position of the mouth corners was not mentioned or in case of posttraumatic or oncologic reconstructions. All case reports and reviews were excluded. Searches were not limited by publication date, language, or publication status (Table 1).

**Table 1.** Inclusion and Exclusion Criteria

Inclusion criteria	Exclusion criteria
Clinical trials	Case reports
Comparative studies	Reviews
Full text available	Letters to editor
All languages	No full text available
Nonsurgical technique(s) to lift the mouth corners	No attention to an effect on the position of the mouth corners nor quantified patient satisfaction
Surgical technique(s) to lift the mouth corners	Posttraumatic reconstruction of the mouth corners
Combination of surgical and nonsurgical technique(s) to lift the mouth corners	(Post)oncologic reconstruction of the mouth corners

### Information Sources and Search

MEDLINE (via PubMed), EMBASE (OvidSP) and the Cochrane Central Register of controlled trials database were searched (searched from May 18, 2019 to December 18, 2021). The search terms (Table 2) were based on two components: (P) corner of the mouth, vermilion border, marionette, oral commissure*, or mouth corner* in combination with (I) surgical procedure*, plastic surgery, esthetic*, operat*, surg*, method*, depressor anguli oris, botulinum toxin, hyaluronic acid, lift*, elevat*, filler*, facial aging, or depress*.

**Table 2.** Specific Search Terms of Databases

Database	Search term
MEDLINE (via PubMed)	((Corner of the mouth[tiab] OR Vermilion border[tiab] OR Marionette[tiab] OR Oral commissure*[tiab] OR Mouth corner*[tiab]) AND (Surgical procedure* OR Plastic surgery OR Esthetic* OR Operat* OR Surg* OR Method* OR Depressor anguli oris OR Botulinum toxin OR Hyaluronic acid OR Lift* OR Elevat* OR Filler* OR Facial aging OR Depress*))
EMBASE (OvidSP)	((“corner of the mouth”:ab,ti OR “vermilion border”:ab,ti OR “marionette”:ab,ti OR oral commissure*:ab,ti OR mouth corner*:ab,ti) AND (surgical procedure*:ab,ti OR “plastic surgery”:ab,ti OR esthetic*:ab,ti OR operat*:ab,ti OR surg*:ab,ti OR method*:ab,ti OR “depressor anguli oris”:ab,ti OR “botulinum toxin”:ab,ti OR “hyaluronic acid”:ab,ti OR lift*:ab,ti OR elevat*:ab,ti OR filler*:ab,ti OR “facial aging”:ab,ti OR depress*:ab,ti)) AND [embase]/lim NOT [medline]/lim AND “article”/it
Cochrane Library	((corner of the mouth OR vermilion border OR marionette OR oral commissure* OR mouth corner*) AND (surgical procedure* OR plastic surgery OR esthetic* OR operat* OR surg* OR method* OR depressor anguli oris OR botulinum toxin OR hyaluronic acid OR lift* OR elevat* OR filler* OR facial aging OR depress*))

### Study Selection and Data Collection Processing

Two of the authors (N.v.d.S. and H.A.G.) performed the search independently. Disagreements were discussed during a consensus meeting. In case of discrepancies between the 2 authors, the senior author (B.v.d.L.) gave a binding verdict.

### Data Items

The search term was based on a PICO framework. Comparisons and outcomes of interest were not included in the search term. For comparisons, we consider the differences in the position of the mouth corners before and after the intervention. In this systematic review, the outcome of interest was the degree of lift and the longevity of the lifting effect, patient satisfaction, and adverse effects. Study characteristics were described.

### Risk of Bias of Individual Studies

Demographics of the included patients were described.

### Risk of Bias Across Studies

The included studies were evaluated for financial support. Disclosure agreements were reviewed for each study.

### Quality Control of Included Studies

The included studies were graded on quality of evidence using the Oxford Center for Evidence-Based Medicine criteria.^27^

## RESULTS

### Included Studies

**Figure 1.**
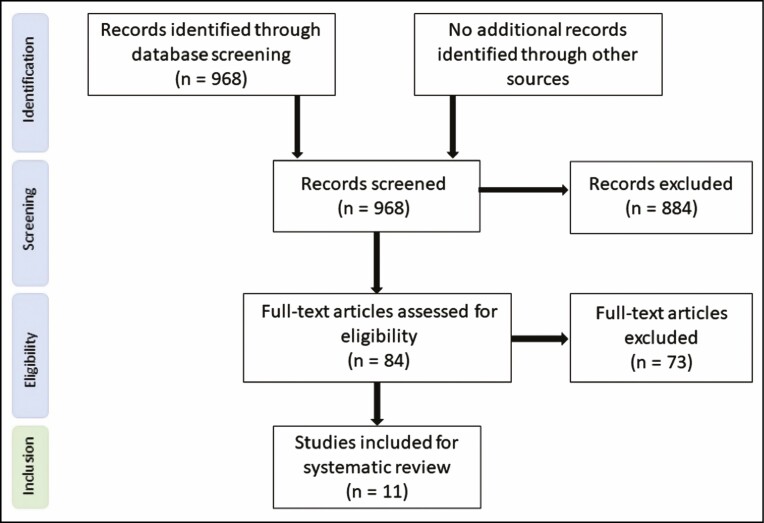
Flow Diagram of Study Selection.

In total, 968 studies were identified after database screening, of which 884 were excluded after abstract screening. Eighty-four full-text studies were assessed on eligibility criteria. Seventy-three studies were excluded because: (1) the effect on the position of the mouth corners was not mentioned, (2) neither the lifting effect of the mouth corner nor patient satisfaction was objectively described (without substantiation of measured values), (3) they were reports of posttraumatic or oncologic reconstructions of mouth corners, (4) they were case reports or review articles, or (5) a combination of the above (Figure 1). One study was published in 2 different journals, and therefore we excluded 1 of these studies.^28,29^

### Study Characteristics

In total, 962 patients were enrolled in the 11 studies.^8,19,20,23,29-35^ Ten studies reported gender of which 95.7% was female (n = 870).^8,19,20,23,29,31-35^ Of the studies enrolled in this systematic review, 6 assessed an invasive technique to lift oral commissures in 426 patients^19,20,23,30-32^ (Supplemental Table 1) and 5 studies assessed a noninvasive technique in 536 patients^8,29,33-35^ (Supplemental Table 2). All studies reported the mean age or age variance of the patients and 7 studies^8,20,23,29,33-35^ described differences in ethnicity or skin type on the Fitzpatrick scale (Supplemental Tables 1 and 2). Four of the 11 included studies were prospective clinical trials,^8,29,33,34^ and all these 4 studies assessed a noninvasive mouth corner lifting technique. No meta-analysis could be performed because the metrics and outcomes were too diverse.

### Invasive Techniques

As for direct surgical techniques to lift oral commissures, 3 studies were included that examined: (1) a simple and an advanced lentiform excision (Parsa et al^20^), (2) a combination of a triangular excision with DAO muscle transection (Pan et al^23^), and (3) an advanced Z-plasty (Kim et al^19^) (Supplemental Table 1). Parsa et al showed a high patient satisfaction rate (>87.5%) and demonstrated good results according to their self-composed assessment scale; however, no objective measurements of lift of the mouth corners were described.^20^ Pan et al used 3-dimensional (3D) photographs to measure the position of the oral commissures before and after treatment and described an effective lift in 78.6% (n = 125) of the patients, accompanied by a patient satisfaction rate of 70.4%.^23^ The extended Z-plasty of Kim et al did result in a significant lift of oral commissure angles measured on 2D photographs. Unfortunately, patient satisfaction was not described in this study.^19^ Notwithstanding, the former studies were all noncontrolled and nonblinded.

As for indirect surgical techniques to lift oral commissures, 3 studies were included that assess: (1) a classical temporal cheek rhytidectomy (McCollough et al^30^), (2) thread lifting (Kaminer et al^31^), and (3) autologous fat grafting (Eremia and Newman^32^) (Supplemental Table 1). McCollough et al assessed the lifting effect on mouth corners in 53 patients after rhytidectomy; an unspecified number of these patients also simultaneously underwent neck lifting. An excellent effect on a 4-point Likert scale was described in 72% (n = 38) of the patients, although 28% (n = 15) of the patients gained minimal to no improvement. However, no objective measurement of lift of the mouth corners or patient satisfaction was described.^30^ Kaminer et al examined the effect of thread lifting on the position of oral commissures in 20 patients, in whom threads were placed in the midface and neck region. Questionnaires based on a 10-point Likert scale showed a patient satisfaction rate of 6.9 out of 10 and an investigator satisfaction rate of 4.6 out of 10. No objective measurements of the lift of the mouth corners were described.^31^ Eremia and Newman assessed the effect of autologous fat grafting on the position of the mouth corners in 116 patients. The 56 patients who were treated for nasolabial folds and oral commissures showed an excellent and stable lift after 3 months. However, this successful mouth corner lift persisted in only 5% (n = 2) of the patients after 12 months, despite additional treatments (up to 3 procedures). No objective measurement of the lift of the mouth corners or patient satisfaction was described in this study.^32^ These studies were all noncontrolled and nonblinded.

### Noninvasive Techniques

Three studies assessed the lifting effect on mouth corners in 484 patients by injecting fillers in perioral tissues^29,33,34^ (Supplemental Table 2). Raspaldo et al used hyaluronic acid (HA) as a filler in 280 patients and showed a high patient satisfaction rate on a 11-point Likert scale, with patients who received Juvederm Volbella (Allergan, Irvine, CA) with lidocaine being significantly more satisfied than patients who received Restylane-L (Galderma, Lausanne, Switzerland). Severity of perioral lines was reduced after 3 months.^29^ D’Aloiso et al used crosslinked carboxymethyl cellulose (CMC) as a filler in 174 patients and demonstrated a patient satisfaction rate of 90.1% after 6 months, measured by >2 points improvement on the Subject Global Aesthetic Improvement Scale (SGAIS); 53% of the patients showed good results on the Marionette Lines Grading Scale (MLGS) after 6 months.^33^ Solish et al used HA (Restylane) as a filler in 30 patients and showed a patient satisfaction after 42 days of good to excellent in 93.3% (n = 28) of the patients, measured on the SGAIS.^34^ Of these studies, the study by Raspaldo et al was the only controlled study^29^ and all studies were nonblinded. All 3 studies describe no objective mouth corner lift after the application of a filler.

One noncontrolled and nonblinded study assessed the effect of injecting Botox in the DAO muscles on the position of the oral commissures in 36 patients^35^ (Supplemental Table 2). Qian et al demonstrated a significant lift of oral commissure angles measured on 2D photographs that was preserved for 6 to 9 months. Unfortunately, patient satisfaction was not described in this study.^35^ One noncontrolled and blinded study assessed the effect of a combination therapy of Botox with HA on the position of the mouth corners in 16 patients^8^ (Supplemental Table 2). Bae et al showed good patient satisfaction in all patients according to the SGAIS and objective measurements of the position of the oral commissures were obtained from 2D photographs. Despite the high patient satisfaction, no statistical differences were found in the median degrees of lifting of the mouth corners 2 weeks and 3 months after treatment.^8^

### Longevity of the Lifting Effect

Six of the 11 studies included a follow-up period of at least 6 months; ^23,29,31-33,35^ 3 of these studies describe an invasive technique and 3 describe a noninvasive technique. A follow up of 12 months or more was described in 2 of the 11 studies.^29,32^ Therefore, a solid foundation to assess the longevity of the lifting effect on the oral commissures is lacking for every technique included in this review.

### Adverse Effects

No severe adverse events were described for both surgical and nonsurgical mouth corner lifting procedures. For both surgical and nonsurgical procedures, minor adverse events such as hematoma and swelling were observed in 14.3% (n = 138) of all patients. For surgical procedures, a scar was inevitable, and an obvious or disturbing scar was observed in 3.8% (n = 16) of all patients.

### Disclosure Agreements

A disclosure agreement of support by the manufacturer, the ministry, or the university was provided in 6 of the 11 studies. ^8,19,23,29,31,34^ If this involved a manufacturer, a different company was involved in all studies (Table 3). Therefore, there was no conflict of interest.

**Table 3.** A Disclosure Agreement of Support by the Manufacturer

Reference	Financial interests or support
Studies using invasive techniques	
Parsa et al, 2010^20^	None reported
Pan et al, 2020^23^	This study was funded by the Interdisciplinary Medicine Seed Fund of Peking University
Kim et al, 2021^19^	This research was supported by a grant of the Korea Health Technology R&D Project through the Korea Health Industry Development Institute (KHIDI), funded by the Ministry of Health & Welfare, Republic of Korea (grant number HI16C2319)
McCollough et al, 2009^30^	None reported
Kaminer et al, 2008^31^	This study was sponsored in part by a grant from Angiotech, Pharmaceuticals, Inc. (Vancouver, British Columbia, Canada)
Eremia and Newman, 2000^32^	No significant financial interest with commercial supporters.
Studies using noninvasive techniques	
Raspaldo et al, 2015^29^	Various authors received research grant support or funding from Allergan, Inc. (Irvine, CA)
D’Aloiso et al, 2016^33^	No significant financial interest with commercial supporters
Solish et al, 2019^34^	Study was funded by Galderma Laboratories, LP (Fort Worth, TX)
Qian et al, 2016^35^	None reported
Bae et al, 2019^8^	This study was supported by research funding from Merz Pharmaceuticals GmbH (Frankfurt, Germany)

### Quality Control of Included Studies

One of the 11 included studies was a Level of Evidence III study,^29^ 6 studies were Level of Evidence IV studies^8,19,23,32-34^ and four studies were Level of Evidence V studies^20,30,31,35^ (Table 4).

**Table 4.** Quality Assessment of Included Studies According to the Oxford Center for Evidence-Based Medicine Criteria

Reference	Level of evidence
Studies using invasive techniques	
Parsa et al, 2010^20^	V
Pan et al, 2020^23^	IV
Kim et al, 2021^19^	IV
McCollough et al, 2009^30^	V
Kaminer et al, 2008^31^	V
Eremia and Newman, 2000^32^	IV
Studies using non-invasive techniques	
Raspaldo et al, 2015^29^	III
D’Aloiso et al, 2016^33^	IV
Solish et al, 2019^34^	IV
Qian et al, 2016^35^	V
Bae et al, 2019^8^	IV

## DISCUSSION

This systematic review demonstrates that most authors publishing about lifting the oral commissures report good patient satisfaction after both surgical and nonsurgical procedures. Surgical procedures seem to have a better lifting effect on mouth corners than nonsurgical procedures, but evidence is weak. However, many techniques do not result in a significant elevation of the corner of the mouth.

Different surgical or invasive techniques to lift the oral commissures have been described in the literature, eg, direct surgical procedures,^4,19-23,36,37^ rhytidectomies,^30,38-40^ thread lifting procedures,^31,41-43^ autologous fat grafting procedures,^32,44,45^ or the insertion of perioral implants.^46,47^ Almost all studies that discuss such techniques claimed a high patient satisfaction; however, most studies failed to evaluate the patient satisfaction through validated questionnaires. Moreover, the effect on the position of the mouth corners was not described in many studies. This systematic review only evaluated those studies that obtained a validated evaluation on patient satisfaction or measured the position of the mouth corners pre- and postoperatively. Our results demonstrate that direct surgical techniques lead to a high effective rate (92%)^23^ and a statistically significant mouth corner lift (*P* < 0.05),^18^ together with good to excellent patient satisfaction. Attending to the underlying mechanism, it is believed that the clinical effect of a surgical treatment is determined by skin excision above the oral commissure, creating a vertical elevation of the mouth corner. Therefore, it is plausible that a skin excision above the corner of the mouth should result in a lifting effect. However, an additional subnasal lift does not lead to a greater lifting effect of the oral commissures.^22^ Only a small group of the patients (3.8%) who underwent a surgical procedure gained an obvious or disturbing scar. The sectioning of the anterior border of the DAO muscles could elevate the position of the corners of the mouth, without involving the smile mechanism.^48^ However, because this review includes no control studies that examine a skin excision with and without a DAO section, we cannot state clearly whether DAO section confers any benefit.

When focusing on indirect surgical procedures, both a rhytidectomy and a thread lifting procedure show no significant lifting effect on the mouth corners. A (mid-)facelift or rhytidectomy is performed to tighten the sagging facial skin of the lower- and midface skin, but the lifting effect or the position of the oral commissures remains unclear. Thread lifting is believed to have a similar tightening effect on lower- and midface skin; however, the more effective the lifting effect, the higher the chance of a “Joker face.” It is believed that the risk of developing a “Joker face” increases when bone anchors are used.^49,50^ Theoretically, even if you do obtain a significant lifting effect on the mouth corner, there is a chance that this effect may disappear as a result of the cheesewire effect after repetitive motion of the mouth and face.^51^ However, because this review included only 1 study that used a thread lifting procedure with a follow-up period of 6 to 16 months, a firm conclusion about the long-term effect cannot be made. It would be interesting if there were more studies investigating the effect on the position of the corners of the mouth after a rhytidectomy or a rhytidectomy combined with a direct surgical mouth corner lift. Autologous fat grafting shows a nonsignificant lifting effect of the mouth corners in the short term (3 months posttreatment) that completely disappeared in the long term (12 months posttreatment, despite additional treatments).^32^ Autologous fat grafting is often used to rejuvenate the dermis; however, the filling effect or regenerating capacity of the adipose stromal cells will not lead to a significant lifting effect on the oral commissures. Nonetheless, all studies that assessed the effect of inserting perioral or lip implants on the rejuvenation of the mouth showed no effect on the position of the oral commissures,^46,47^ therefore these studies were excluded from this systematic review.

Noninvasive techniques to rejuvenate perioral tissues or lift the mouth corners include injecting fillers, Botox, or a combination of these. Fillers around the mouth corners have been used to provide structure and support in this area to lift the corners of the mouth.^52^ This systematic review evaluated 3 studies in which a monotherapy with HA or CMC was applied to rejuvenate or lift the corners of the mouth.^29,33,34^ All studies showed good to high patient satisfaction rates; however, no significant lifting effect on the oral commissures was shown. The fact that most patients were satisfied with a mouth lifting procedure, despite an objective lifting effect, is probably due to a certain “sham” effect.

Since the introduction and use of Botox for treating wrinkles, Botox has also been applied to rejuvenate oral commissures.^53,54^ The aim of applying Botox to lift oral commissures is to inhibit the action of muscles that lead to drooping of the mouth corners when activated, eg, DAO muscles. Different types of Botox (eg, onabotulinumtoxin-A, abobotulinumtoxin-A, and incobotulinumtoxin-A) in varying doses have been used for nonsurgical elevation of the corner of the mouth by injecting the DAO muscles. Furthermore, different variations or extended injection techniques have been described, eg, the “Nefertiti lift” (involving injection of neurotoxins into platysmal bands and the inferior border of the mandible).^55,56^ Claude le Louarn stated that frequently blocking the DAO muscles is compensated by the contraction of the platysma,^48^ and therefore injecting Botox into both DAO muscles and platysmal bands might result in a more effective mouth corner lift. However, more research is necessary to corroborate this. This systematic review evaluated 1 study (n = 36) in which injecting botulinum toxin type A into DAO muscles resulted in a significant lift of the mouth corner, which persisted for approximately half a year.^35^ The longevity of this effect is interesting, especially because it is believed that the effects of botulinum toxin type A wear off about 3 to 4 months after injection.^57^ Although the objective evidence is weak, we assume that the application of Botox might be a safe, noninvasive, but temporary technique to lift mouth corners. This technique needs to be repeated to obtain a lasting effect. In addition to this, a combination therapy of a filler with Botox shows high patient satisfaction rates without a real mouth corner lifting effect, corresponding to the effects of a monotherapy with fillers.

Out of the 968 articles we found after database screening, only 11 studies met the inclusion criteria for this review. The main reasons for this limited number of articles were that most articles fail to pay specific attention to the position of the oral commissures or lack measurable results. In addition, the reported gender in the included studies was predominantly female (95.7%), and therefore it cannot be determined with certainty whether gender affects outcomes. Furthermore, the reported mean ages vary greatly and because aging has an effect on the position of the corners of the mouth, age could affect (long-term) outcomes. We recommend that future studies should validate the lifting effect on the position of the oral commissures and objectively measure patient satisfaction, to better investigate the outcome of a surgical or a nonsurgical mouth corner lifting technique.

## CONCLUSIONS

This systematic review is the first study to have evaluated both surgical and nonsurgical mouth corner lifting procedures in terms of their efficacy, patient satisfaction, and adverse effects. Overall, surgical techniques seem to produce a better and more sustainable lifting effect on mouth corners than nonsurgical techniques; however, objective evidence is weak. Injecting Botox into DAO muscles could be a scarless but temporary alternative to a surgical lift.

## Supplementary Material

sjac077_suppl_Supplementary_Tables
