# Correction to: Lifting the Mouth Corner: A Systematic Review of Techniques, Clinical Outcomes, and Patient Satisfaction

**DOI:** 10.1093/asj/sjae079

**Published:** 2024-04-16

This is a correction to: “Lifting the Mouth Corner: A Systematic Review of Techniques, Clinical Outcomes, and Patient Satisfaction,” by Nanouk van der Sluis, Haydar A Gülbitti, Joris A van Dongen, and Berend van der Lei, published in *Aesthetic Surgery Journal*, Volume 42, Issue 8, August 2022, Pages 833–841, https://doi.org/10.1093/asj/sjac077.

In this article, co-author Dr Nanouk van der Sluis was incorrectly listed in the affiliations section as a professor, however, his correct title at the time of publication was “surgical resident.” The intent of this correction notice is to clarify the correct job title for Dr van der Sluis for the purpose of correcting the academic record for this article. The authors appreciate the opportunity to correct this information.

This information is reflected within this correction notice in order to officially document the correction to the article while preserving the published version of record.

