# Supplementary material for: Lifting the Mouth Corner: A Systematic Review of Techniques, Clinical Outcomes, and Patient Satisfaction
Source: Aesthet Surg J. 2022 Apr 4;42(8):833–41. doi: 10.1093/asj/sjac077 (PMC9342679; doi:10.1093/asj/sjac077)
Supplement: sjac077_suppl_Supplementary_Tables [file sjac077_suppl_supplementary_tables.docx]

**S1 Table.** Study Characteristics, Patient Demographics, Results, and Adverse Effects of 6 Studies Using Invasive Techniques

| Reference | Technique | Study type | Number of patients (n) | Gender | | Age (yr) | | Ethnicity | Fitzpatrick skin type | Follow-up | Lift of mouth corners | | Patient satisfaction | Adverse effects |
| --- | --- | --- | --- | --- | --- | --- | --- | --- | --- | --- | --- | --- | --- | --- |
|  |  |  |  | Male | Female | Mean ± SD | Range |  |  |  | |  |  |  |
| Parsa et al, 2010 ^20^ | Lentiform excision (simple (1) and advanced including marionette lines (2)). | Retrospective, non-controlled, non-blinded, non-randomized, cohort study | 27 | 3 | 24 | 61.3 (type 1) and 69.1 (type 2) | N/A | Caucasian and Asian (or mixed) | N/A | 12 months (n=24) and 3 months (n=3) | The grades of 1, 2, and 3 were given to “very noticeable,” “moderately noticeable,” and “barely noticeable/not noticeable” scars, respectively.  Good results were described according to a self-composed assessment scale.  No objective measurement of a mouth corner lift was described. | | > 87.5% of the patients were satisfied according to a self-composed questionnaire. | Depressed scars (1.9%, n=2) and superficial wound infection (3.7%, n=1) were described. |
| Pan et al, 2020 ^23^ | Combined method of triangular excision with DAO transection. | Retrospective, non-controlled, non-blinded, non-randomized, observational study | 159 | 1 | 158 | 26.7 ± 5.2 | 18-53 | Asian | N/A | 6-72 months | 3-Dimensional measurement showed that the average postoperative width of oral fissure was 45.1±3.2 mm and the average postoperative difference between the horizontal height of Point 1 (the mouth corner) and Point 2 (10 mm medial of the ipsilateral mouth corner at caudal edge of the upper lip) was 0.9 ± 0.5 mm.  Effective (78.6%, n=125) to mildly effective (13.2%, n=21) results were described, however a small part (8.2%, n=13) was ineffective.  The effective rate [(obviously effective + mildly effective)/total case] for objective evaluation was 92%. | | 70.4% of the patients were satisfied according to a self-composed questionnaire.  5.0% (n=8) were unsatisfied (total score <70 for ten items, in the items those scored <4, 6/8 were “incision scar”, 4/8 were “vermilion shape”, 4/8 were “naturality”, and 1/8 were “marionette lines occurrence”). | The most common short-term complication was inflammatory reaction to sutures (15.7%, n=25). The most common long-term complications were obvious incision scars (5.7%, n=9) and bilateral asymmetry (5%, n=8). |
| Kim et al, 2021 ^19^ | Z-plasty with excision of skin and subcutaneous tissue, cutting the fibrous connection between the modiolus and the corner of the OOM and tiding the corner of the OOM to the anterior border of the LAOM. | Retrospective, non-controlled, non-blinded, non-randomized, observational study | 51, of which 33 received a facelift combined with an oral commissure lift and 18 received solely an oral commissure lift | 3 | 48 | 46.7 ± 11.9 | N/A | N/A | N/A | 25.2 ± 22.9 months | The preoperative mean angles of the right and left oral commissures measured - 3.1 ± 4.0° and - 3.4 ± 3.7°, respectively, and postoperative mean angles measured 3.6 ± 3.2° and 3.3 ± 3.5°, respectively. Postoperative changes in oral commissure angles were statistically significant (P<0.05). | | Patient satisfaction was not described. | Visible scarring (4%, n=2), undercorrection (2%, n=1) and asymmetry (2%, n=1) were described. |
| McCollough et al, 2009 ^30^ | Classical temporal cheek rhytidectomy.  Many patients also underwent neck-lifting simultaneously (exact number not mentioned). | Retrospective, non-controlled, non-blinded, non-randomized, observational study | 53 | N/A | N/A | 57 | N/A | N/A | N/A | 2 – 23 months | 72% (n=38) of the patients gained an excellent effect on a 4-point Likert scale. 28% (n=15) of the patients gained minimal to no improvement.  Reviewers of obtained pre- and postoperative photographs included 1 plastic surgeon, 1 cosmetic surgeon and 1 facial plastic surgery clinical fellow.  No objective measurement of a mouth corner lift was described. | | Patient satisfaction was not described. | Adverse effects were not described. |
| Kaminer et al, 2008 ^31^ | Barbed suture threads placed in the midface and neck region (6-8 per side). | Retrospective, non-controlled, non-blinded, non-randomized, case-series | 20 | 0 | 20 | 58.3 | 39-73 | N/A | N/A | 6 – 16 months | When evaluating pre- and postoperative photographs for individual facial zones, a higher average improvement was observed in the nasolabial zone (average, 4.8; SD, 0.68) and the marionette lines/jowls zone (average, 4.6; SD, 0.92), compared to the neck zone (average, 3.9; SD, 1.18).  No objective measurement of a mouth corner lift was described. | | Questionnaire with a 10-point Likert scale (1=completely unsatisfied, 10=completely satisfied). 12 out of 20 patients returned surveys. Self-reported patient satisfaction was rated 6.9/10.  Preoperative and postoperative photographs of 7 patients were assessed by 7 independent dermatologists, that rated the overall improvement an average of 4.6/10. | More than half of the patients experienced adverse effects, e.g. bruising (58%, n=7), swelling (54%, n=6), pinching sensation (27%, n=3), visibility of threads (36%, n=4) and ear numbness (25%, n=3). However, no intervention was required. |
| Eremia and Newman, 2000 ^32^ | Autologous fat grafting session(s), whereby the amounts injected were ie,.1.5-2.5 cc (nasolabial fold) and 1-1.5 cc (per lateral oral commissure).  All patients received prophylactic antibiotics. | Retrospective, non-controlled, non-blinded, non-randomized, observational study | 116 patients underwent a total of 307 treatment sessions | 12 | 104 | 57 | 34-72 | N/A | N/A | > 12 months | After 3 months,  all 56 patients that were treated (1x) for nasolabial folds and oral commissures, were rated A (excellent and stable lift).  After 12 months, of the 52 patients that were treated (3x) for nasolabial folds and oral commissures, 2 (5%) were rated A, 5 (11%) were rated B (visible correction compared to baseline, about 50 ± 20% loss of correction between what would be considered “A” and baseline) and 45 (84%) were rated C (no lift or completely depressed to baseline).  No objective measurement of a mouth corner lift was described. | | Patient satisfaction was not described. | Hematoma at the donor site (3.3%, n=10), scarring at the recipient site (0.98%, n=3) that needed revision and temporary asymmetry (0.3%, n=1) were observed.   Edema, postoperative discomfort, and minor hematoma at the recipient sites, which resolved within a few days, were not considered as complications. |

DAO(M), depressor anguli oris (muscle); OO(M), orbicularis oris (muscle); LAO(M), levator anguli oris (muscle); SD, standard deviation.

**S2 Table.** Study Characteristics, Patient Demographics, Results, and Adverse Effects of 5 Studies using Non-invasive Techniques

| Reference | Technique | Study type | Number of patients (n) | Gender | | Age (yr) | | Ethnicity | Fitzpatrick skin type | Follow Up | Lift of mouth corners | Patient satisfaction | Adverse effects |
| --- | --- | --- | --- | --- | --- | --- | --- | --- | --- | --- | --- | --- | --- |
|  |  |  |  | Male | Female | Mean ± SD | Range |  |  |  |  |  |  |
| Raspaldo et al, 2015 ^29^ | HA (Juvederm Volbella with lidocaine (‘A’) compared to Restylane-L (‘B’)) injected in peri-oral tissues.  Mean volume per oral commissure was 0.60 mL per injectable.  The maximum allowable treatment volume per patient was 4.0 mL, including top up therapy (n=30, 21,4%). | Prospective, controlled, non-blinded, randomized, 2-arm, multicenter study | 280, of which 139 received ‘A’ and 141 received ‘B’.  126 patients received injections in oral commissures with ’A’ and 124 patients with ‘B’ | 4 (‘A’)  2 (‘B’) | 135 (’A’)  139 (’B’) | 48 (‘A’)  49 (’B’) | 18 – 76 (‘A’)  18 – 75 (’B’) | N/A | Most patients had skin type II or III | 2 months | Both treatments reduced the severity of perioral lines and oral commissures at month 3.  No objective measurement of a mouth corner lift was described. | All subjects were satisfied to a 11-point Likert scale, whereby patients that received ‘’A’’ were significantly more satisfied at month 3 than patients that received ‘’B’’ (P<0.05). | Small adverse effects, such as lumps and firmness were described in 10.1%, n=4 (‘’A’’) respectively 13.5%, n=5 (‘’B’’) of subjects. No serious adverse effects were described. |
| D’Aloiso et al, 2016 ^33^ | CMC filler injected (tiny amounts) in the mid-deep dermis of peri-oral tissues. | Prospective, non-controlled, blinded, non-randomized, open-label, pilot clinical study | 174, of which 86 received injections in marionette lines | 9 | 165 | 52 | N/A | N/A | Most patients had skin type III (69%) and II (21%) | 6 months | MLGS at 3 months and at 6 months respectively score 0 to 1 in 61% procedures (54/86) and in 53% (47/86) (P<0.001). Median grade of amelioration of MLGS was 2±1 and 87% of procedures classified as 0 to 1 at 3 months and maintained the same at 6 months.  No objective measurement of a mouth corner lift was described. | Three months postoperative, 92% of patients self-reported a significant improvement (>2 SGAIS). After 6 months 90.1% of patients remained the same amount of improvement. | Temporary small ecchymosis (8%, n=14) was described. |
| Solish et al, 2019 ^34^ | Hyaluronic Acid (HA, Restylane)  in peri-oral tissues.  The recommended maximum injection volume per session was  1 mL per ML. | Prospective, non-controlled, non-blinded, non-randomized, open‐label, pilot clinical study | 30 | 0 | 30 | 55 | 40 - 65 | Caucasian | N/A | 42 days | All subjects showed at least a 1‐grade improvement in ML severity bilaterally based on WAS at day 42 compared with baseline. 83.3% (n=25) had at least a 2‐grade improvement. The mean (SD) change from baseline in bilateral WAS scores at Day 42 was −2.15 (0.59) (P<0.001).  No objective measurement of a mouth corner lift was described. | Subjects reported high levels of satisfaction with their aesthetic outcome at day 42 using a 5‐point Likert scale.  SGAIS showed “very much improved” at day  42 for 40% (n=12) of patients, 53.3% (n=16) ‘’much improved’’ and 6.7% (n=2) ‘’somewhat improved’’. | Almost all patients experienced bruising, swelling and redness (97.6%, n=29). |
| Qian et al, 2016 ^35^ | BoTox (botulinum toxin A, 2-4 U/site)  in DAOM bilaterally. | Retrospective, non-controlled, non-blinded, non-randomized, observational study | 36 | 3 | 33 | 28.6 | 23 - 35 | Asian | N/A | 6 – 12 months | When the mouth corner (points A and B) was located below the horizontal line of the mouth orifice, the degree of mouth corner drooping was defined as positive;  when points A and B were located above the horizontal line of the mouth orifice, the degree of mouth corner drooping was defined as negative.  Significant lifting effect 1 month after application was measured using the paired t test (P<0.01). This effect was preserved for 6 –9 months. | Patient satisfaction was not described. | No patients experienced adverse effects. |
| Bae et al, 2019 ^8^ | A combination of 1) BoTox (incobotulinum toxin A, 16U) and 2) Monophasic HA.  injecting 1) in the DAO and mentalis muscle .  injecting 2) in the mouth corner and upper and lower vermillion borders. | Prospective, non-controlled, blinded, non-randomized , pilot clinical study | 16 | 2 | 14 | 40 | 24 – 71 | Asian | N/A | 3 months | Frontal photographs were taken at baseline, day 0, 2 weeks and 3 months after treatment. The degree of drooping of the mouth corners was  measured at each side of the mouth at baseline  No statistical differences  were found in the median degrees of drooping of the mouth corners  at 2 weeks and 3 months after treatment (P>0.05). | All patients were satisfied according to the SGAIS. | There were no adverse effects described. |

DAO(M), Depressor Anguli Oris Muscle; HA, Hyaluronic Acid; BoTox , Botulinum toxin; (C)CMC, Cross-linked CarboxyMethyl Cellulose; ML, Marionette Line; (S)GAIS, (Subject) Global Aesthetic Improvement Scale; MLGS, Marionette Lines Grading Scale; WAS, Wrinkle Assessment Scale; SD, Standard Deviation.
